# *In Vitro* Expansion and Characterization of Mesenchymal Stromal Cells from Peritoneal Dialysis Effluent in a Human Protein Medium

**DOI:** 10.1155/2018/5868745

**Published:** 2018-10-03

**Authors:** Bo La Han, Lan Zhou, Qiunong Guan, Gerald da Roza, Hao Wang, Caigan Du

**Affiliations:** ^1^Department of Urologic Sciences, University of British Columbia, Vancouver, BC, Canada; ^2^Department of Urology, East Hospital, Tongji University, Shanghai, China; ^3^Division of Nephrology, Department of Medicine, University of British Columbia, Vancouver, BC, Canada; ^4^Department of General Surgery, Tianjin Medical University General Hospital, Tianjin, China; ^5^Tianjin General Surgery Institute, Tianjin, China

## Abstract

The therapeutic potential of mesenchymal stromal cells (MSCs) from various tissue origins have extensively been explored in both experimental and clinical studies, and peritoneal dialysis effluent-derived MSC (pMSC) may be an easily obtainable MSC source for clinical applications. In this study, we expanded and characterized the pMSCs after expansion in a human protein culture medium. The pMSCs were expanded in plastic dishes with the human protein medium. MSC marker expression was examined by flow cytometry. Spherical formation was tested by hanging drop method, and osteogenic, adipogenic, and chondrogenic differentiation capacities were confirmed by positive staining with Alizarin red, Oil red O, and Alcian blue, respectively. Here, we showed that after four passages of culturing in plastic dishes, pMSCs in the human protein medium displayed a homogeneous pattern of classical MSC markers (positive: CD29, CD44, CD73, CD90, and CD166; negative: CD14, CD34, CD45, CD79a, CD105, CD146, CD271, HLA-DR, SSEA-4, and Stro-1), while in the standard medium, pMSCs from some donors were CD45 or HLA-DR positive. For nonclassical MSC markers, pMSCs were CD200 positive from all the donors, negative for CD163, CD271, CD36, and CD248, and either positive or negative for CD274 and CD140b. Further, pMSCs from the human protein medium had the spherical formation capacity and multipotent differentiation capacity *in vitro*. In conclusion, upon expansion in a human protein medium, pMSCs showed a differential MSC marker expression profile from those of bone marrow or adipose tissue-derived MSCs and could maintain the multipotency. The therapeutic potential of the pMSCs requires further investigation.

## 1. Introduction

Mesenchymal stromal cells (MSCs) are self-renewal, multipotent, fibroblast-like adult cells that have been found in a variety of adult tissues in our body, such as the bone marrow (BM), adipose tissue, lung, peripheral blood, umbilical cord blood, placenta, and fetal tissues [1]. Since the first time Friedenstein and his colleagues identified MSCs in the BM in 1976 [2], the therapeutic potential of a MSC-based therapy has been widely explored in treatment of a variety of human diseases, particularly in the field of both regenerative medicine and immunotherapy [3, 4]. Indeed, currently, more than 800 clinical trials have been registered at the American National Institute of Health (clinicaltrials.gov) aimed at evaluating the therapeutic potential of MSCs worldwide.

BM-derived MSCs (BM-MSCs) are the most extensively studied source of MSCs, but the low yield (0.001%–0.01%) and invasive aspiration procedure greatly hinder the clinical use of BM-MSCs [5]. Both embryonic and aborted fetal tissues can provide a high yield of MSCs [6–8]; however, the use of these sources of MSCs for clinical applications and research is limited due to ethical and safety issues. Therefore, finding alternative sources of MSC is crucial in the current development of a MSC-based therapy. Our group, for the first time, has identified and isolated MSCs in peritoneal dialysis (PD) effluent from PD patients [9]. With its large source of otherwise discarded PD effluent, which can easily be collected from PD clinics, the PD effluent-derived MSCs (pMSCs) may be a feasible alternative source of MSCs for the MSC therapy [9].

As *in vivo* sources usually do not provide enough number of MSCs, one of the requirements for successful development of the MSC-based therapy is having an effective culture method for *in vitro* expansion, to obtain a large quantity of clinically graded MSCs [10]. Currently, the standard *in vitro* expansion of MSCs uses a fetal bovine serum- (FBS-) containing culture medium, in which FBS provides nutrients and attachment factors for cell growth and attachment [11]. However, the clinical use of animal-derived (xeno) products such as FBS poses several safety concerns [11–13]. For instance, xeno antigens and infectious agents in FBS may be transmitted to the MSC recipients [11]. Therefore, careful use of or avoiding such xeno products in the process of MSC expansion is an important aspect in the clinical translation of the MSC therapy [11, 12, 14]. The objective of this study was to characterize pMSCs in a xeno-free human protein-based culture system.

## 2. Materials and Methods

### 2.1. PD Effluent Collection

PD effluents were collected from anonymized donors as described by Liu et al. [9], which was approved by the Clinical Research Ethics Board at the University of British Columbia (Vancouver, BC) in accordance with the Canadian Tri-council policy statement: ethical conduct for research involving humans (protocol number: H15-02466). A total of ten PD effluent samples were collected from ten donor patients (one from each) who were on PD therapy with either Dianeal or Physioneal PD solution within 4 weeks (Table 1).

### 2.2. Antibodies

The following fluorescent-conjugated monoclonal antibodies were used in this study: rat allophycocyanin (APC) anti-human/mouse CD44 (clone IM7, *e*Bioscience, San Diego, CA, USA), APC mouse anti-human CD34 (clone 4H11, *e*Bioscience), fluorescein isothiocyanate (FITC) mouse anti-human Stro-1 (clone MOPC-104E, BioLegend, San Diego, CA, USA), phycoerythrin (PE) mouse anti-human CD146 (clone P1H12, BD Biosciences, Mississauga, ON, Canada), APC mouse anti-human CD29 (clone TS2/16, BioLegend), FITC mouse anti-human CD90 (Thy-1) (clone eBio5E10, *e*Bioscience), FITC mouse anti-human HLA-DR (clone L243, *e*Bioscience), PE mouse anti-human CD79a (clone HM47, *e*Bioscience), PE mouse anti-human CD166 (ALCAM) (clone 3A6, *e*Bioscience), APC mouse anti-human CD14 (clone 61D3, *e*Bioscience), FITC mouse anti-human CD105 (Endoglin) (clone 266, BD Biosciences), APC mouse anti-human CD45 (clone H130, BD Biosciences), PE mouse anti-human CD271 (clone C40-1457, BD Biosciences), FITC mouse anti-SSEA-4 (clone MC813-70, BD Biosciences), PE mouse anti-human CD73 (clone AD2, BD Biosciences), PE mouse anti-human CD36 (clone CD38, BD Biosciences), PE mouse anti-human CD 140b (clone 28D4, BD Biosciences), PE mouse anti-human CD 274 (clone MIH1, BD Biosciences), FITC mouse anti-human CD 163 (clone GHI/61, BD Biosciences), Alexa Fluor 647 mouse anti-human endosialin CD 248 (clone B1/35, BD Biosciences), and PE-Cy™ mouse anti-human CD 200 (clone MRC Ox-104, BD Biosciences).

### 2.3. Isolation of Adherent Cells and Proteins from PD Effluent

The isolation of both adherent cells and proteins from PD effluent was processed within 12 h after collection from donors. Adherent cells were isolated from the effluents and cultured, as described previously by Liu et al. [9]. In brief, cells in PD effluents were pelleted by centrifugation at 751 ×g at 10°C for 10 min. After washing with phosphate-buffered saline (PBS, pH 7.4), the resultant cells were suspended and cultured in plastic culture dishes with Dulbecco's modified Eagle's medium/Ham's nutrient mixture F12 (DMEM/F12, 50/50) containing either 10% human protein solution (v/v), as described below, or FBS and 1% penicillin/streptomycin solution (100×, Thermo Fisher Scientific, Mississauga, ON, Canada) at 37°C in a 5% CO_2_ atmosphere incubator.

After the cell isolation, proteins were isolated from PD effluents for preparation of a human protein-based culture system for *ex vivo* expansion of pMSCs. In brief, after pelleting the adherent cells from the PD effluents as described above, the supernatant was further centrifuged at a high speed (8000 **×**g), in order to remove cell debris. Then, the proteins were precipitated by addition of ammonium sulphate (to 80% saturation) at room temperature (RT), and the precipitated protein fraction was pelleted from the solution by centrifugation at 48000 **×**g for 20 min (Avanti J-E centrifuge with JLA-16.25 rotor, Beckman Coulter Inc., Mississauga, ON, Canada). The protein pellets were dissolved in distilled water, and the remaining ammonium sulphate in the solution was removed by dialysis in distilled water, using a cellulose tubular membrane (Cellu-Sep T2/Nominal MWCO: 6000-8000, Genprice Inc., San Jose, CA, USA) for three days with daily water change. After a three-day dialysis, the protein content was measured by using Bio-Rad protein assay (absorption at 595 nm), and the optical density (OD) reading at 595 nm of the human protein solution was approximately 1. The stock protein solution was kept at −25°C until it was used in the culture medium.

### 2.4. Flow Cytometric Analysis of Cell Surface Markers

After four passages (P4) of culturing cells in plastic dishes, the expression levels of panels of both classical and nonclassical MSC surface markers were examined using fluorescence-activated cell sorting (FACS) analysis, as described by Liu et al. [9]. In brief, after a short trypsinization of the adherent cells from the plastic dishes using Trypin-EDTA solution (Sigma-Aldrich Canada, Oakville, ON, Canada), a single cell suspension was prepared by suspending cells in a culture medium. Then, the cells were incubated with each type of the antibodies as listed above in the dark for 30 min at 4°C. After washing with PBS, the fluorescence intensity of the stain was counted using a Calibur flow cytometer (BD Biosciences). Data were analyzed with the FlowJo software (FlowJo LLC, Ashland, OR, USA).

### 2.5. Trilineage Differentiation Assays

The chondrogenic differentiation was performed using a high-density cell culture, as described by Liu et al. [9]. In brief, after the cells have been cultured to P4, 10 *μ*L droplets of cells (1 × 10^6^/mL) were placed on a 10 cm petri dish and incubated at 37°C for 2 h, followed by incubation in high glucose DMEM medium supplemented with 1% (v/v) human proteins, 10 ng/mL transforming growth factor-*β*1 (Sigma-Aldrich Canada), 50 *μ*g/mL ascorbate acid, 0.1 *μ*M dexamethasone, 100 *μ*g/mL sodium pyruvate, 40 *μ*g/mL proline, and 50 mg/mL ITS premix (5 *μ*g/mL insulin, 5 *μ*g/mL transferrin, and 5 ng/mL selenious acid). The cultures were then maintained for 4 weeks, and the medium was changed twice a week. After 4 weeks of incubation, the cells were stained with 1% of acidic Alcian blue in 80% methanol (v/v) (pH 2.5). The positive results indicated chondrogenic differentiation or the presence of cartilage formation.

The osteogenic or adipogenic differentiation was induced in confluent cultures in plastic culture dishes after P4 according to protocol described by Liu et al. [9]. For osteogenic differentiation, the cells (1 × 10^6^ cells/well in 6-well plates) were treated with high glucose DMEM supplemented with 10% (v/v) human proteins, 50 *μ*g/mL ascorbic acid, 10 nM dexamethasone (Sigma-Aldrich Canada), 10 mM *β*-glycerol phosphate (Sigma-Aldrich Canada), and 3.7 mg/mL sodium bicarbonate for 4 weeks. The culture medium was changed twice a week. After the 4-week incubation period, the osteogenic differentiation was confirmed by staining Ca^2+^ matrix mineralization with 2% Alizarin red S in 0.5% NH_4_OH (pH 4.2). For adipogenic differentiation, the cells (1 × 10^6^ cells/well in 6-well plates) were incubated with high glucose DMEM supplemented with 10% (v/v) human proteins, 5 nM hydrocortisone, 50 *μ*g/mL ascorbic acid, 50 *μ*g/mL indomethacin, and 1 *μ*M dexamethasone. The culture medium was changed twice a week for 4 weeks. The adipogenic differentiation has been examined by the presence of lipid droplets which are stained with 0.14% Oil red O following the protocol in Lonza website (http://www.lonza.com).

### 2.6. Determination of Spheroid Formation *In Vitro*

The spheroid formation of adult pMSCs *in vitro* was determined by using hanging drop cell culture technique as described previously [15]. In brief, after P4, 0.25 × 10^6^ cells in a volume of 10 *μ*L were dropped onto the bottom of the lid of a tissue culture dish. Around 10 droplets were placed per dish, with sufficient rooms between the droplets. The lid was inverted onto a PBS-filled bottom chamber and incubated in a humidified 37°C/5% CO_2_ incubator. Spheroids were observed under a light microscope after approximately 2–5 days of incubation and were separated by gentle shaking.

## 3. Results

### 3.1. Morphology and Cell Surface Markers of pMSCs from the Human Protein-Based Culture System

**Figure 1:**
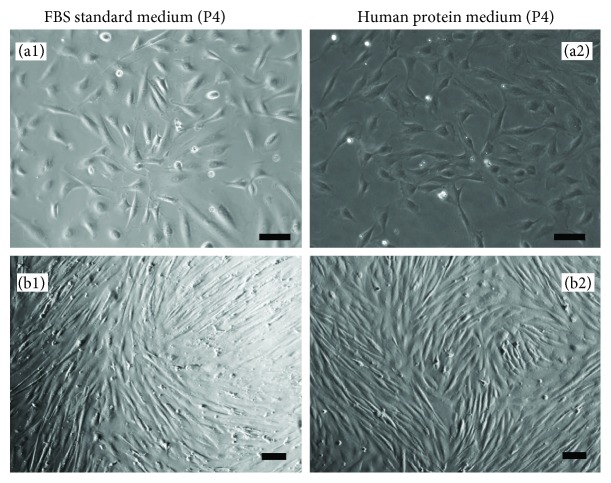
A typical microscopic view of adherent pMSC morphology after expansion in human protein medium. Adherent cells from PD effluent of a donor were divided into two parts: one growing in FBS-containing standard medium and the other in human protein medium. After four passages (P4) in both media, unsorted cells in the plastic culture dishes displayed a typical morphology of mesenchymal cells. (a1, a2) Low-density cells; (b1, b2) high-density or confluent cells. Scale bar, 100 *μ*m. Data were representative of five donors.

**Figure 2:**
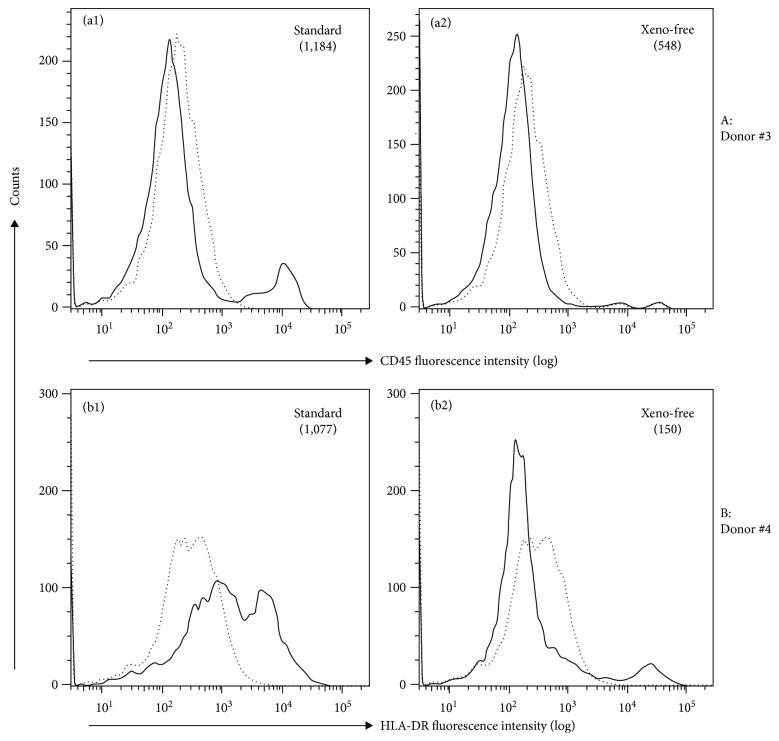
A different expression of CD45 or HLA-DR of pMSCs after expansion between standard medium and human protein medium. The different expression of a panel of classical MSC markers in unsorted pMSCs was compared after P4 between FBS-containing standard medium and in human protein-based xeno-free medium. Histograms of FACS analysis showed CD45 positive in pMSCs from donor #3 after expansion in the standard medium but not in the xeno-free medium (a1, a2), HLA-DR positive in pMSCs from donor #4 in the standard medium but not in the xeno-free medium (b1, b2). Dotted line: background fluorescence intensity of a control antibody, solid line: fluorescence intensity of anti-CD45 or anti-HLA-DR antibody staining.

One of the challenges in clinical translation of MSC therapies is to have basic characterization of the MSC product [16]. In this study, we characterized the expression of common MSC surface markers in pMSCs after expansion in the human protein-based (xeno-free) culture medium. We compared the expression of MSC markers on the pMSC after expansion between the xeno-free human protein culture medium and in FBS standard medium. We randomly selected five donors for this study (Table 1, donor #1 to #5); the cell pellets from PD effluent of each donor were split into two parts; one part was grown in the standard FBS medium and the other in human protein (xeno-free) medium. The cell growth rates were not significantly different between these two different media at each passage (data not shown). After P4, all of the cells were plastic-adherent and exhibited spindle or fibroblastoid morphology in plastic culture dishes (Figure 1). The cell surface markers of these adherent cells were compared at P4. As listed in Table 2, the adherent cells from all of the five donors in the xeno-free medium had a similar expression profile of the following cell surface markers: positive expression of CD29, CD44, CD73, CD90, and CD166 and negative expression of CD14, CD34, CD45, CD79a, CD105, CD146, CD271, HLA-DR, SSEA-4, and Stro-1. In the standard FBS medium, although a similar expression pattern was seen (Table 2), positive expression of CD 45 was seen in the cells from donor #3 (Figure 2(a), Table 2) and of HLA-DR from donor #4 (Figure 2(b), Table 2).

**Figure 3:**
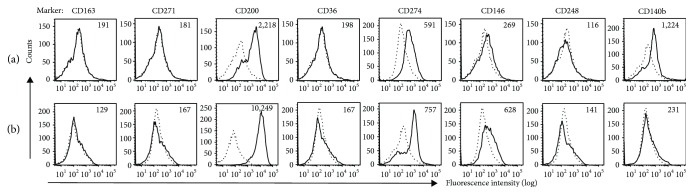
Histograms of nonclassical MSC marker expression in pMSCs from selected donors. The expression of a panel of nonclassical MSC markers in unsorted pMSCs from different donors was investigated after P4 in a human protein-based xeno-free medium. Histograms of FACS analysis showed the positive expression of CD200, CD274, and CD140b in pMSCs from donor #6 (a) and the positive expression of CD200, CD274, and CD146 in those from donor #10 (b). The rest of nonclassical markers were negative. Dotted line: background fluorescence intensity of a control antibody, solid line: fluorescence intensity of an anti-non-classical MSC marker antibody staining.

To further compare the cell surface MSC markers of pMSCs with other types of MSCs, expression of a panel of nonclassical MSC markers in pMSCs was examined after expansion in the human protein medium. The nonclassical MSC markers were identified in adipose-derived MSCs (AMSCs) grown in human platelet lysate (hPL) culture medium [17] and included CD163, CD271, CD200, CD36, CD274, CD146, CD248, and CD140b. As shown in Figure 3 and Table 3, CD200 was the only marker that was strongly expressed in the pMSCs from all of the five randomly selected donors (Table 1, donor #6 to donor #10), and there were some weak expressions of CD274 and CD140b in donor #6 (Figure 3(a)) and CD274 and CD146 in donor #10 (Figure 3(b)). The rest of the markers (CD163, CD271, CD36, and CD248) were negative in pMSCs (Figure 3, Table 3).

### 3.2. Multipotential of pMSCs from the Human Protein-Based Culture System for Trilineage Differentiation

**Figure 4:**
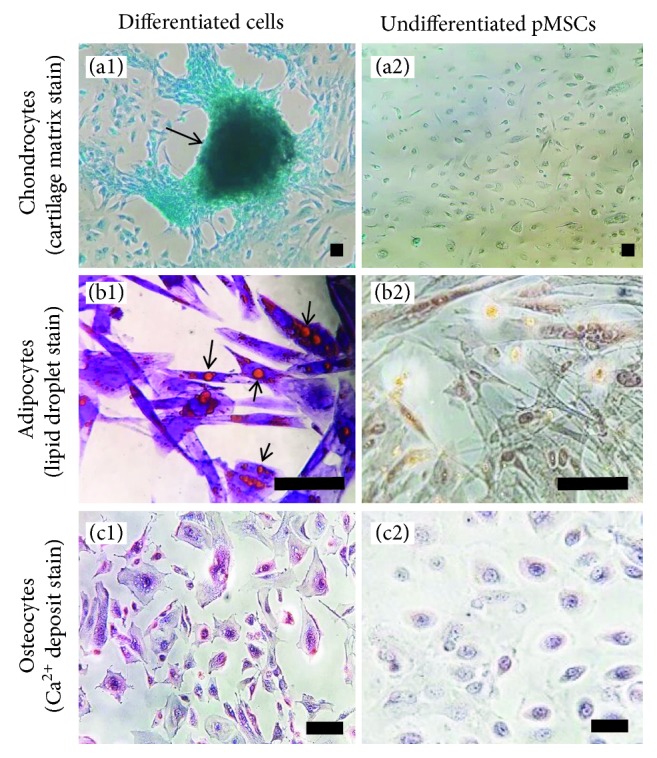
A typical microscopic view of differentiated cell staining. Adherent cells from PD effluent were grown in plastic culture plates with human protein medium. After P4, unsorted pMSCs were induced to chondrocytes, adipocytes, or osteocytes by incubation with each different differentiation medium for 4 weeks. The nondifferentiated pMSCs were collected without incubation with the differentiation medium. (a1, a2) Differentiation to chondrocytes, both differentiated and nondifferentiated cells were stained with Alcian blue. Arrow: cartilage matrix in “clustering” cells. (b1, b2) Differentiation to adipocytes, both differentiated and nondifferentiated cells were stained with Oil red O. Arrows: lipid droplets. (c1, c2) Differentiation to osteocytes, both differentiated and nondifferentiated cells were stained with Alizarin red S. Red: extracellular calcium deposits. Scale bar, 100 *μ*m. Data were presented as trilineage differentiation of representative pMSCs from ten donors.

One of the biological properties of adult MSCs is their multipotent differentiation capacity that is an important aspect for clinical application in regenerative medicine [18]. In this study, we confirmed the trilineage differentiation capacity after expansion in human protein culture medium *in vitro* as compared with those in standard FBS medium. Following incubation with differentiation media, specific for each cell type, the pMSCs that had been expanded in the human protein medium underwent differentiation into chondrocytes, adipocytes, or osteocytes in the same manner as pMSCs expanded in the standard FBS medium [9]. Chondrogenic differentiation was demonstrated by Alcian blue staining of cartilage matrix in the cells that formed colonies (Figure 4(a)), adipocyte differentiation by Oil red O staining of the lipid droplets in the differentiated cells (Figure 4(b)), and osteocyte differentiation by Alizarin red S staining, showing the presence of extracellular calcium deposits (Figure 4(c)). No staining was seen in those control undifferentiated pMSC cultures—grown in the xeno-free medium only (without induction of differentiation with the differentiation media). The differentiation capacity of pMSCs from this xeno-free culturing system was not different from those from the standard FBS cultures.

### 3.3. Spheroid Formation of pMSCs from the Human Protein-Based Culture System

**Figure 5:**
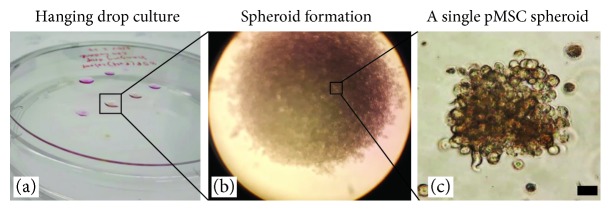
A typical microscopic view of a pMSC spheroid culture After P4 in human protein medium, unsorted pMSCs were growing in a hanging drop culture (a). After 5 to 7 days of incubation, many spheroids were generated (b) and were easily separated by gentle shaking (c). Scale bar, 100 *μ*m. Data were presented as representative pMSCs of ten donors.

One approach to optimize MSC preparation for the cell therapy is generation of MSC spheroids [19]. In this study, we examined the *in vitro* spheroid formation capacity of pMSCs after expansion in the human protein medium. After P4 in plastic dishes with the human protein medium, cells were harvested and their capacity of forming spheroids was tested by incubation in hanging drop cultures. As shown in Figure 5, pMSCs from the xeno-free culture system formed a significant number of spheroids after 5 to 7 days of incubation, suggesting that these pMSC spheroids could be prepared in a large quantity for future clinical applications [20].

## 4. Discussion

The therapeutic potentials of MSCs have been widely explored in many preclinical and clinical studies. Recent meta-analysis of clinical trials supports the clinical benefits of MSCs on treatment of osteoarthritis [21], Crohn's disease [22], acute myocardial infarction [23], and chronic liver disease [24]. However, a robust and reproducible manufacturing of clinical-graded MSCs in a xeno-free condition is required for wide application of the MSC therapy [20]. We were the first group to identify pMSCs in otherwise discarded PD effluent that may become an alternative source of MSCs [9]. In this follow-up study, we have demonstrated that these pMSCs can be expanded in a human protein medium without alteration of MSC morphology, cell surface phenotype, and *in vitro* trilineage differentiation. Also, the isolated pMSCs were able to expand in spheroids, which suggests the possibility of manufacturing pMSC spheroids in a large batch for future clinical applications.

The International Society for Cellular Therapy (ISCT) has recommended minimal criteria for identifying MSCs, which included plastic-adherence, trilineage differentiation capacity (osteogenic, adipogenic, and chondrogenic), positive expression of CD105, CD73, and CD90, and negative expression of CD45, CD34, CD14 or CD11b, CD79a or CD19, and HLA-DR [25]. Similar to the minimal criteria proposed by ISCT, a recent systemic review summarized common MSC surface markers in literatures [26], in which the most common positive markers of adult MSCs are CD29, CD44, CD90, and CD105, while the most prominent negative markers are CD14, CD34, and CD45 [26]. Our studies have demonstrated that pMSCs from all donors express all the positive markers (CD29, CD44, CD73, and CD90), except CD105 that is negative in both FBS and human protein culture media (Table 1) [9]. Similar to pMSCs, the human skin-derived MSCs are negative in the expression of CD105 [27], and the lack of CD105 does not affect the chondrogenic potential of human BM-MSCs [28]. Together, all these data may suggest that CD105 may not be a necessary marker for MSCs from the different sources. Furthermore, pMSCs were negative in the expression of CD14, CD34, and CD45 after expansion in the human protein culture medium (Table 2), which are also listed as top negative markers for MSCs in literatures [26]. Similar to CD105, Stro-1 was negative in pMSCs in both FBS standard and human protein culture conditions (Table 2) [9]. Again, Stro-1 is positive in the BM-MSCs [26], adipose tissues [29], and dental pulp [30] but was negative in pMSCs in our studies. In the comparison between pMSCs and AMSCs in the expression of nonclassical cell surface markers, Camilleri et al. [17] have reported that the AMSCs positively express CD36, CD146, CD248, CD140b, and CD274 and lack of expression of CD163, CD271, and CD200, but in the present study, pMSCs are CD200-positive and CD248-negative (Table 3). Taken together, the expression of some cell surface markers (i.e., CD105, Stro-1, CD200, and CD248) in pMSCs is different from that in other types of MSCs, such as BM-MSCs and AMSCs. This finding may suggest that pMSCs have unique origin, different from BM or adipose tissues.

Mechanisms of how different cell surface molecules (markers) regulate biological functions of MSCs are not fully understood. Unlike the AMSCs [17], pMSCs consistently expressed CD200 (OX-2 membrane glycoprotein) which is absent/expressed at a low level in AMSCs. Interestingly, CD200 expression is associated with adipogenic capacity and, the expression declines in AMSCs during adipogenesis [31]. Since CD200 expression is inversely correlated with adipogenesis, the level of CD200 may reflect innate adipogenic capacity of MSCs. For example, since AMSCs require high adipogenic capacity *in vivo* in order to replenish cells from adipose tissues, the expression of CD200, which is inversely correlated to adipogenesis, is kept low. Thus, low level of CD200 in AMSCs and high level of CD200 in pMSCs may suggest that CD200 expression is tissue-specific and depends on the need from the source. Moreover, pMSCs did not express CD36 and CD248, both of which are expressed in AMSCs [17]. It further addresses that CD 36 is a highly specific marker for AMSCs [17]. CD 248 (endosialin), on the other hand, is known to play various roles in MSCs such as negative regulation of bone formation and thymus remodeling and regeneration [32, 33]. We also observed weak expression of CD274 (B7H1/PDL1), CD140b (PDGFRB), and CD146 with greater variabilities between donors (Table 3). The functional roles of CD274 in pMSCs are not yet investigated; however, CD274 has been shown to be involved in immunosuppressive roles in BM-MSCs [34–37]. The expression of CD146 is associated with vascular smooth muscle commitment in BM-MSCs and defines a subpopulation of BM-MSCs that are capable of differentiating towards osteogenesis *in vitro* and bone formation *in vivo* [38, 39]. CD140b (PDGFRB) is involved in the recruitment of MSCs and enhances the development and repair of stromal tissue types [40], but its expression is MSC type-dependent, positive in AMSCs, endometrium-derived MSCs (eMSCs), and perivascular MSC from human brain but negative in BM-MSCs [17, 40–42].

In mammalian cell cultures, FBS is commonly added to a culture medium to support cell growth and attachment. However, in clinical cell therapies, the use of FBS should be avoided as it poses several safety concerns, such as disease-induced antigens [43, 44]. We compared the expression of cell surface markers of pMSCs expanded in the human protein medium with those cells expanded in FBS-containing medium (Table 2) [9]. It was interesting to note that weak expression of three typical hematopoietic markers (CD14, CD45, and HLA-DR) was found in some donors after expansion in the standard FBS medium (Table 2) [9], but none of these hematopoietic markers was expressed in the cells from the same donors after expansion in the human protein medium (Table 2). These data may suggest the possibility of the presence of monocytes/macrophages in pMSCs expanded in the FBS medium, and/or some undefined xenogeneic components from FBS may stimulate the expression of CD14, CD45, or HLA-DR in pMSCs from some donors, which, however, requires further investigation.

In addition to the need to use a xeno-free cell culture system, optimization of MSC preparation in a large quantity is also urgently required [45]. Production of MSC spheroids represents one method of the optimization of MSC preparation for MSC therapies [19, 20], by which the anti-inflammation, multilineage differentiation potential, and survival of MSCs after transplantation are enhanced [19]. Following the standard protocol, the adherent cells from PD effluent after P4 were capable of being expanded in a 3D culture condition in spheroidal cell aggregates (Figure 5), which may reflect the capability of manufacturing large quantity of pMSC spheroids, optimal for clinical use. However, the significance of pMSC spheroids in their biological activities requires further investigation. Currently, we are testing the therapeutic efficacy of pMSCs after *ex vivo* expansion in the xeno-free human protein medium in a rat model of PD. Our preliminary data show that infusion of these pMSCs back to the peritoneal cavity protects the peritoneal membrane from hypertonic PD solution-induced tissue damage (manuscript in preparation).

The limitations of the present study can be found as follows. Firstly, we only sampled a limited number of donors for each experiment due to time constraints and sample availability; thus, the results could be strengthened more with additional donors. Secondly, the human proteins we tested were purified from PD effluent, and their donor-to-donor variability remains unknown. Finally, the biological activities of pMSCs from plastic petri dishes or in spheroids were not examined as compared with other well-studied MSCs such as BM-MSCs or AMSCs.

## 5. Conclusion

It has previously been suggested that pMSCs isolated from otherwise discarded PD effluent may serve as an alternative source for MSCs for a cell therapy [9]. In this study, we demonstrated that pMSC may serve as a unique alternative source with its distinct expression patterns of both classical and nonclassical MSC markers after expansion in a human protein medium. In addition to the differences in the expressions of CD 105, Stro-1, and SSEA-4 in pMSCs from BM-MSCs, pMSC expressed CD200, CD248, and CD36 differentially from AMSCs. We also highlighted the potential for the clinical translation of pMSC-based therapy by demonstrating the expansion of pMSC in a 3D spheroid culture condition.

## Figures and Tables

**Table 1:** The demographic information of donors.

Donor no.	Age (yr)	Gender (F/M)	Race/ethnicity	Time on PD (wk)	PD solution
1	65	M	Caucasian	1	Physioneal
2	49	M	Asian	3	Physioneal
3	83	M	Caucasian	2	Physioneal
4	62	M	Caucasian	1	Dianeal
5	57	F	Latin American	2	Physioneal
6	60	M	Caucasian	4	Dianeal
7	60	M	Caucasian	3	Dianeal
8	71	M	Asian	4	Physioneal
9	53	M	Asian	2	Dianeal
10	60	F	Asian	3	Physioneal

The PD effluents were collected under protocol H15-02466 approved by the Clinical Research Ethics Board at the University of British Columbia.

**Table 2:** Comparison of classical MSC marker expression of pMSCs after P4 in FBS standard medium with that after P4 in human protein (xeno-free) medium.

Donor no.	1		2		3		4		5	
Medium	Standard	Xeno-free	Standard	Xeno-free	Standard	Xeno-free	Standard	Xeno-free	Standard	Xeno-free
CD14	214	117	155	110	207	140	189	156	135	101
CD29	16,435	27,096	27,172	49,100	48,154	70,848	50,064	38,239	45,174	40,875
CD34	143	135	153	162	230	190	106	163	192	104
CD44	34,600	16,572	20,185	39,866	63,599	53,580	21,398	43,814	50,905	50,764
CD45	147	151	132	117	1184	548	135	158	175	178
CD73	1126	1265	1563	5656	1500	5382	519	1941	2444	2424
CD79a	145	164	171	136	535	246	248	128	123	189
CD90	17,962	15,247	8252	10,723	14,105	14,383	8391	19,469	23,128	36,576
CD105	189	203	198	142	443	228	532	238	253	244
CD146	377	196	243	156	394	246	148	144	199	169
CD166	704	495	530	409	1462	2944	567	1611	5019	4895
CD271	144	161	164	133	282	215	92.4	158	126	186
HLA-DR	138	143	176	141	329	127	1077	150	182	189
SSEA	163	24.1	224	117	334	137	301	119	210	195
Stro-1	158	17.6	194	126	338	135	279	134	203	222

Data were presented by median of fluorescence intensity (MFI). Background MFI: 224 ± 50.7.

**Table 3:** Nonclassical MSC marker expression of pMSCs after P4 in human protein (xeno-free) medium.

Donor no.	6	7	8	9	10
CD163	191	262	143	138	129
CD271	181	242	233	175	167
CD200	2218	5547	834	11,809	10,249
CD36	198	218	160	179	167
CD274	591	394	241	469	757
CD146	269	328	246	475	628
CD248	116	232	116	138	141
CD140b	1224	254	407	337	231

Data were presented by median of fluorescence intensity (MFI). Background MFI: 224 ± 50.7.

## Data Availability

All the data used to support the findings of this study are included within the article and are also available from the corresponding author (Caigan Du) upon request.
